# Validation of the Toronto hepatocellular carcinoma risk index for patients with cirrhosis in China: a retrospective cohort study

**DOI:** 10.1186/s12957-019-1619-3

**Published:** 2019-04-30

**Authors:** Huixian Zhang, Jinzhou Zhu, Liting Xi, Chunfang Xu, Airong Wu

**Affiliations:** Department of Gastroenterology, The First Affiliated Hospital of Soochow University, 188 Shizi Street, Suzhou, 215000 Jiangsu China

**Keywords:** Cirrhosis, Hepatocellular carcinoma (HCC), Toronto hepatocellular carcinoma risk index (THRI), Validation

## Abstract

**Background:**

The Toronto hepatocellular carcinoma (HCC) risk index (THRI) was developed to predict HCC in patients with cirrhosis. This study aimed to validate the THRI in a 10-year Asian cohort.

**Methods:**

A total of 2836 patients with cirrhosis at the First Affiliated Hospital of Soochow University between January 2008 and May 2018 were evaluated. Based on the THRI value at diagnosis, patients were divided into three groups (< 120, low-risk; 120–240, intermediate-risk; > 240, high-risk). Student’s *t* test and Fisher’s exact test were applied to compare parameters between the HCC group and the non-HCC group. The receiver operator characteristic (ROC) curve was drafted to identify the value of the THRI in predicting HCC. Logistic regression was utilized to assess the relationship between the development of HCC and THRI values. The incidence of HCC was calculated for the three groups using the Kaplan-Meier method, and curves were compared using the log-rank test.

**Results:**

Of 520 patients enrolled in this study, 76 patients developed HCC. Patients who developed HCC had a higher THRI score than those who did not develop HCC (279.5 ± 57.1 vs. 232.3 ± 67.6, respectively, *p* < 0.001). The area under the ROC curve for the THRI to predict HCC was 0.707 ([95% CI 0.645–0.769], *p* < 0.001), with a sensitivity of 0.842 and a specificity of 0.486 when the cutoff THRI value was 226. Compared to the low-risk group, the high-risk group presented higher odds of developing HCC (adjusting odds ratio 1.026 [95% CI 1.002–1.051], *p* = 0.036). Differences existed in the cumulative incidence of HCC among the three risk groups (log-rank, *p* < 0.001). The 5-year cumulative HCC incidence of the low-risk group, intermediate-risk group, and high-risk group was 0%, 13%, and 34%, respectively.

**Conclusion:**

This study validated THRI values for predicting HCC in Asians with cirrhosis, which presented a fine sensitivity to identify the high-risk population of HCC for secondary prevention.

**Electronic supplementary material:**

The online version of this article (10.1186/s12957-019-1619-3) contains supplementary material, which is available to authorized users.

## Background

Hepatocellular carcinoma (HCC) is the seventh most common cancer and the fourth leading cause of cancer-related deaths in the world [1]. Approximately 75–85% of HCC cases were reported in Asian countries each year, while China alone accounted for 55% of HCC cases worldwide [2]. Because the majority of patients with HCC are diagnosed at advanced stages [3], few curable treatments can be applied. Even worse, spontaneous rupture of advanced HCC is fatal and accelerates the advent of poor outcomes [4, 5].

Population-based screening promotes the early identification of high-risk patients for developing HCC [6–8]. Both the American Association for the Study of Liver Diseases (AASLD) and the Asian Pacific Association for the Study of the Liver (APASL) suggested a combination of alpha-fetoprotein (AFP) level and ultrasound as monitoring tools for HCC, and a surveillance interval of 6 months was recommended for patients with chronic hepatitis and HBV carriers [9, 10].

Patients infected with HBV were the major population at risk of HCC. In China, HBV infection is predominant [11], with approximately 69 million people infected, 120 million carriers and 20 million people with chronic hepatitis [12]. Several studies revealed that surveillance for high-risk people with HBV-related liver disease offered an opportunity for early detection and improved the rate of curative treatment [13, 14]. Several scoring systems and models were developed to predict the risk of HCC, including the Chinese University of Hong Kong (CUHK) clinical scoring system [15]; the Risk Evaluation of Viral Load Elevation and Associated Liver Disease/Cancer-Hepatitis B Virus (REVEAL-HBV) nomograms [16]; the Guide with Age, Gender, HBV DNA, Core Promoter Mutations and Cirrhosis (GAG-HCC) risk score [17]; the Risk estimation for hepatocellular carcinoma in chronic hepatitis B (REACH-B) [18]; the Hepatitis C Antiviral Long-Term Treatment Against Cirrhosis (HALT-C) model [19]; and the Age, Diabetes, Race, Etiology of cirrhosis, Sex and Severity of liver dysfunction (ADRESS)-HCC risk model [20].

The incidence of HCC differed strikingly among disease etiologies. The risk of developing HCC in patients infected with HBV and/or HCV increased on the basis of established cirrhosis or advanced fibrosis [10]. The scoring systems mentioned above are mainly HBV- and/or HCV-related and were developed mainly among cohorts of patients with hepatitis B and/or hepatitis C infection. Most of them had either failed to take multiple cirrhosis etiologies into account or lacked validation experiments of large samples in multiple regions. The ADRESS-HCC model [20] was developed to predict the 1-year risk of HCC in patients with cirrhosis in the US, yet only three broad categories of etiology (autoimmune, alcohol/metabolic, and viral) were included in the final model. A comprehensive model is urgently needed to predict the risk of HCC.

In 2017, experts from Canada and Europe used readily available clinical and laboratory parameters and developed a risk index, the Toronto hepatocellular carcinoma risk index (THRI)[21], to predict HCC in patients with cirrhosis of various categories, which helped to screen high-risk populations and improve the secondary prevention of HCC. Since the spectrum of HCC differs between Western and Eastern regions, this retrospective cohort study aimed to validate the THRI for predicting HCC in Asian patients with different types of cirrhosis.

## Methods

### Population

The digital records of a total of 2836 patients with cirrhosis treated at the First Affiliated Hospital of Soochow University during the past 10 years were used in this study. This study was approved by the Ethics Committee of the First Affiliated Hospital of Soochow University.

Positive evidence of imaging examinations, such as B-ultrasound, computed tomography (CT), and magnetic resonance imaging (MRI), together with pathological evidence, was taken as confirmatory evidence of cirrhosis. Clinical manifestations, including varices, variceal hemorrhage, and ascites, were used as identified evidence when portal hypertension and ascites caused by other diseases were excluded.

During the follow-up, each participant had at least two visits (every 6 months) for imaging examinations (ultrasound, CT, and MRI) with or without AFP test, which were recorded in Additional file 1 in detail.

Diagnosis of HCC was consistent with APASL guidelines and specifications for diagnosis and treatment of primary hepatocellular carcinoma in China [10, 22]. Positive evidence of CEUS, dynamic CT, dynamic MRI, gadolinium ethoxybenzyl diethylenetriamine pentaacetic acid (Gd-EOB-DTPA)-enhanced MRI (EOB-MRI) and diagnostic biopsy were important evidence to diagnose HCC.

The inclusion criteria included the following: (1) diagnosis of cirrhosis between January 2008 and May 2018; (2) confirmatory evidence of imaging features of cirrhosis on ultrasound, CT, and MRI; clinical symptoms caused by cirrhosis; and (3) adherence to at least six monthly follow-ups.

**Fig. 1:**
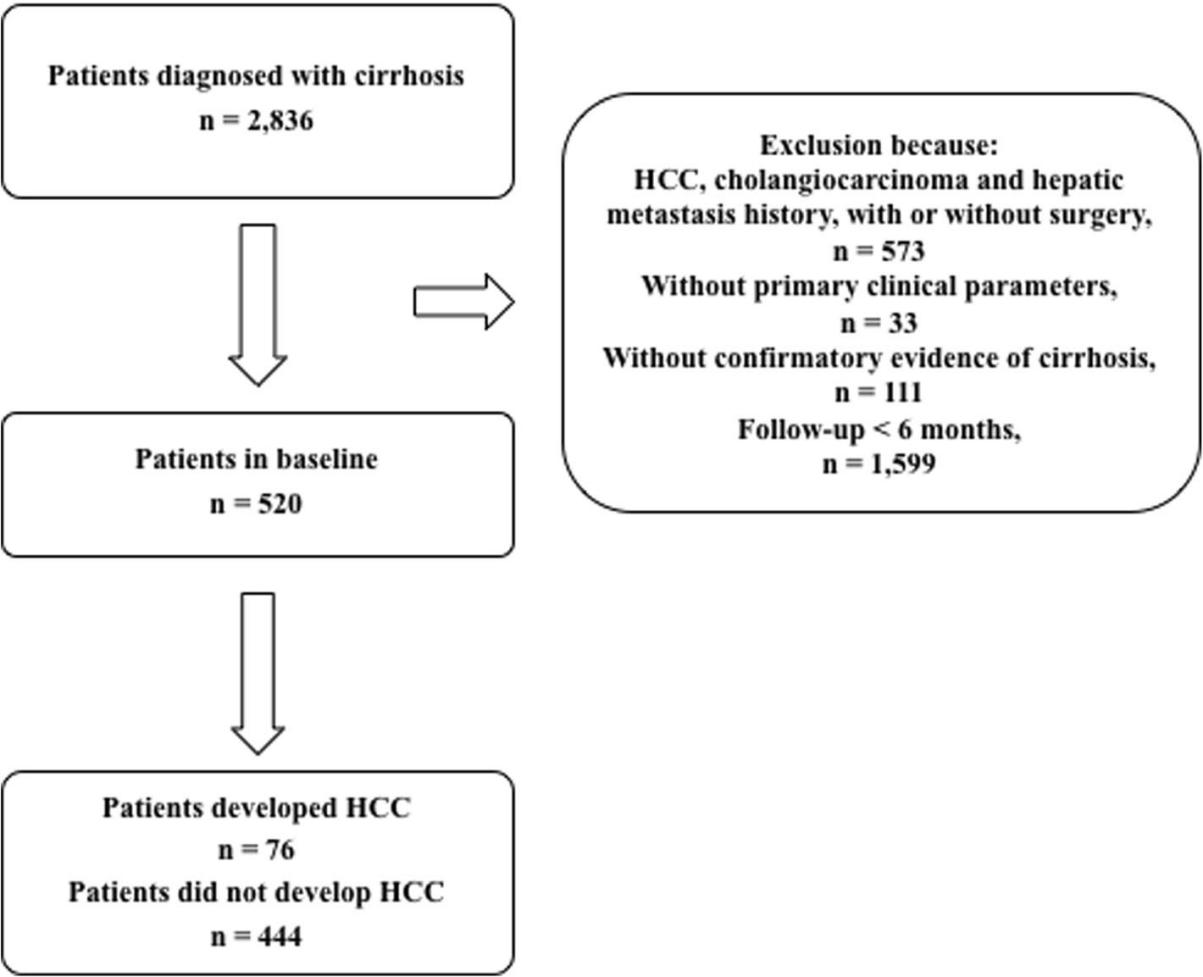
Flow chart of selecting participants. HCC hepatocellular carcinoma

The exclusion criteria included the following: (1) pre-existing HCC, cholangiocarcinoma and metastatic liver cancer history, with or without surgery (hepatectomy or liver transplant therapy); (2) without primary clinical parameters or alternative data within 3 months; (3) unconfirmed evidence of cirrhosis; and (4) follow-up for < 6 months (*n* = 1599) (Fig. 1).

Cirrhosis caused by chronic hepatitis B (CHB) and chronic hepatitis C (CHC) was defined as the “HBV group” and “HCV group,” respectively. Cirrhosis developed from steatohepatitis was distributed into the “alcoholic liver disease (ALD) group” and the “non-alcoholic fatty liver disease (NAFLD) group.” Autoimmune liver diseases included autoimmune hepatitis (AIH), primary biliary cirrhosis (PBC), and primary sclerosing cholangitis (PSC) and were represented in three groups with the same names. The “Other group” consisted of cryptogenic cirrhosis, cirrhosis caused by circulatory objection, medicine, parasitic infection, and genetic and metabolic diseases, such as Wilson disease, hereditary hemochromatosis, and alpha-1 antitrypsin deficiency.

The time of initial visit and last follow-up (for patients who developed HCC, their last follow-up was defined as the time of HCC diagnosis), together with physical and clinical parameters (platelet count, prothrombin time, international normalized ratio [INR], alanine transaminase [ALT], aspartate aminotransferase [AST], bilirubin, albumin, etc.), was recorded. In addition, AST to platelet ratio index (APRI) score, Fibrosis-4 (FIB-4) score, model for end-stage liver disease (MELD) score, and Child-Pugh score were calculated for each patient.

### THRI

The THRI assigned weighted values to four risk factors (age, etiology, sex, and platelets) and evaluated HCC risk of cirrhosis with different etiologies. Patients were stratified into three risk groups based on the THRI score (low-risk as < 120, intermediate-risk as 120–240 and high-risk as > 240).

### Statistical analysis

Continuous data were demonstrated as the mean ± standard deviation (SD) if normally distributed and the median (interquartile range [IQR]) if nonnormal. Student’s *t* test and *χ*^2^ test (Fisher’s exact test) were applied to compare demographic and clinical parameters between the HCC group and the non-HCC group. The receiver operator characteristic (ROC) curve was drafted to identify the value of the THRI in predicting HCC risk and to define the optimal cutoff point for predicting HCC risk. Logistic regression was chosen to assess the relationship between HCC development and the THRI. Considering that body mass index (BMI) and other clinical parameters might act as potential cofounders, logistic regression models were utilized. The incidence of HCC was calculated for the low-risk, intermediate-risk, and high-risk groups using the Kaplan-Meier method, with curves compared using the log-rank test. The HCC incidence of different cirrhosis etiologies was also compared in the same way. All the statistical analyses and plotting were performed using SPSS (version 21.0, SPSS, Inc., Chicago, IL, USA) and Stata (version MP11.2, Stata Corp LP, College Station, TX, USA). A *p* value of less than 0.05 was considered statistically significant.

## Results

### Identification of the cohort

In total, 2836 patients were admitted to the First Affiliated Hospital of Soochow University who were diagnosed with cirrhosis from January 2008 to May 2018; however, 2316 patients were excluded from the study (Fig. 1). A total of 520 patients were enrolled in this study.

### Baseline characteristics

The baseline characteristics of the patients are shown in Table 1. Five hundred and twenty patients met the inclusion criteria of our study, with a mean age of 60.5 ± 13.0 years. The mean duration of the follow-up was 32.4 ± 23.2 months. Apart from the Other group, CHB (*n* = 184) was the main cause of cirrhosis, followed by AIH (*n* = 33), ALD (*n* = 30), PBC (*n* = 23), CHC (*n* = 8), NAFLD (*n* = 1), and PSC (*n* = 1). There was no HCV-SVR group because none of the eight patients with CHC achieved SVR. Four of them did not receive antiviral therapy due to poor liver function and physical condition. The rest received the treatment of PR (Pegylated interferon-alfa plus Ribavirin) with or without DAAs (Direct-acting antiviral agents) discontinuously owing to intolerance of medicine and serious adverse reactions. Therefore, all of the eight patients remained viremic.

**Table 1:** Baseline characteristics

	All patients (*n* = 520)	HCC (*n* = 76)	Non-HCC (*n* = 444)	*p* value (HCC vs. non-HCC)
Mean age ± SD, years	60.46 ± 12.95 (24–89)	59.76 ± 11.67 (36–84)	60.58 ± 13.16 (24–89)	0.613
Sex				0.000
Female, *n* (%)	219 (42)	16 (21)	203 (46)	
Male, *n* (%)	301 (58)	60 (79)	241 (54)	
Mean follow-up ± SD, months	32.48 ± 23.22	39.57 ± 25.90	31.27 ± 22.54	0.004
Median follow-up (range), months	26.86 (6.18–109.48)	32.30 (6.77–104.02)	25.59 (6.18–109.48)	
Etiology, *n* (%)				0.000
HBV	184 (35)	46 (61)	138 (31)	
HCV	8 (2)	3 (4)	5 (1)	
ALD	30 (6)	6 (8)	24 (5)	
NAFLD	1 (0)	0 (0)	1 (0)	
AIH	33 (6)	2 (3)	31 (7)	
PBC	23 (4)	0 (0)	23 (5)	
PSC	1 (0)	0 (0)	1 (0)	
OTHER	240 (46)	19 (25)	221 (50)	
Mean BMI ± SD, kg/m^2^	23.27 ± 3.44	23.68 ± 3.13	23.18 ± 3.51	0.359
Mean PLT ± SD, 10E9/L	99.86 ± 69.27	87.99 ± 66.83	101.90 ± 69.55	0.106
Mean INR ± SD	1.28 ± 0.39	1.31 ± 0.23	1.27 ± 0.41	0.512
Mean PT ± SD, sec	14.96 ± 4.98	15.44 ± 2.70	14.87 ± 5.28	0.396
Mean ALT ± SD, U/L	60.49 ± 152.66	61.44 ± 161.54	60.32 ± 151.29	0.954
Mean AST ± SD, U/L	68.15 ± 124.32	65.24 ± 97.63	68.66 ± 128.45	0.826
Mean GGT ± SD, U/L	113.52 ± 141.60	89.04 ± 90.89	117.81 ± 148.37	0.107
Mean ALB ± SD, g/L	33.09 ± 6.33	33.00 ± 6.01	33.10 ± 6.39	0.897
Mean TBil ± SD, μmol/L	38.98 ± 48.44	35.24 ± 30.86	39.61 ± 50.81	0.476
Mean Cr ± SD, μmol/L	75.08 ± 54.49	73.65 ± 33.97	75.31 ± 57.16	0.816
Mean AFP ± SD, μg/L	14.38 ± 59.90	24.85 ± 51.65	12.31 ± 61.29	0.227
Mean THRI ± SD	239.17 ± 68.21	279.49 ± 57.07	232.27 ± 67.62	0.000
Mean APRI ± SD	2.40 ± 4.07	3.04 ± 7.65	2.29 ± 3.06	0.138
Mean FIB-4 ± SD	7.71 ± 6.31	8.58 ± 6.44	7.56 ± 6.28	0.197
Mean MELD ± SD	7.57 ± 5.99	7.81 ± 6.53	7.53 ± 5.90	0.731
Mean Child-Pugh Score ± SD	7.15 ± 1.74	7.28 ± 2.09	7.13 ± 1.67	0.545

*SD* standard deviation, *HBV* hepatitis B virus, *HCV* hepatitis C virus, *ALD* alcohol liver disease, *NAFLD* non-alcoholic fatty liver disease, *AIH* autoimmune hepatitis, *PBC* primary biliary cirrhosis, *PSC* primary sclerosing cholangitis, *BMI* body mass index, *PLT* platelet count, *PT* prothrombin time, *INR* international normalized ratio, *ALT* alanine aminotransferase, *AST* aspartate aminotransferase, *GGT* γ-glutamyl transpeptidase, *ALB* albumin, *TBil* total bilirubin, *Cr* creatinine, *AFP* alpha fetoprotein, *THRI* Toronto hepatocellular carcinoma risk index, *APRI* AST to platelet ratio index, *FIB-4* Fibrosis-4 score, *MELD* model for end-stage liver disease

### THRI and HCC

**Fig. 2:**
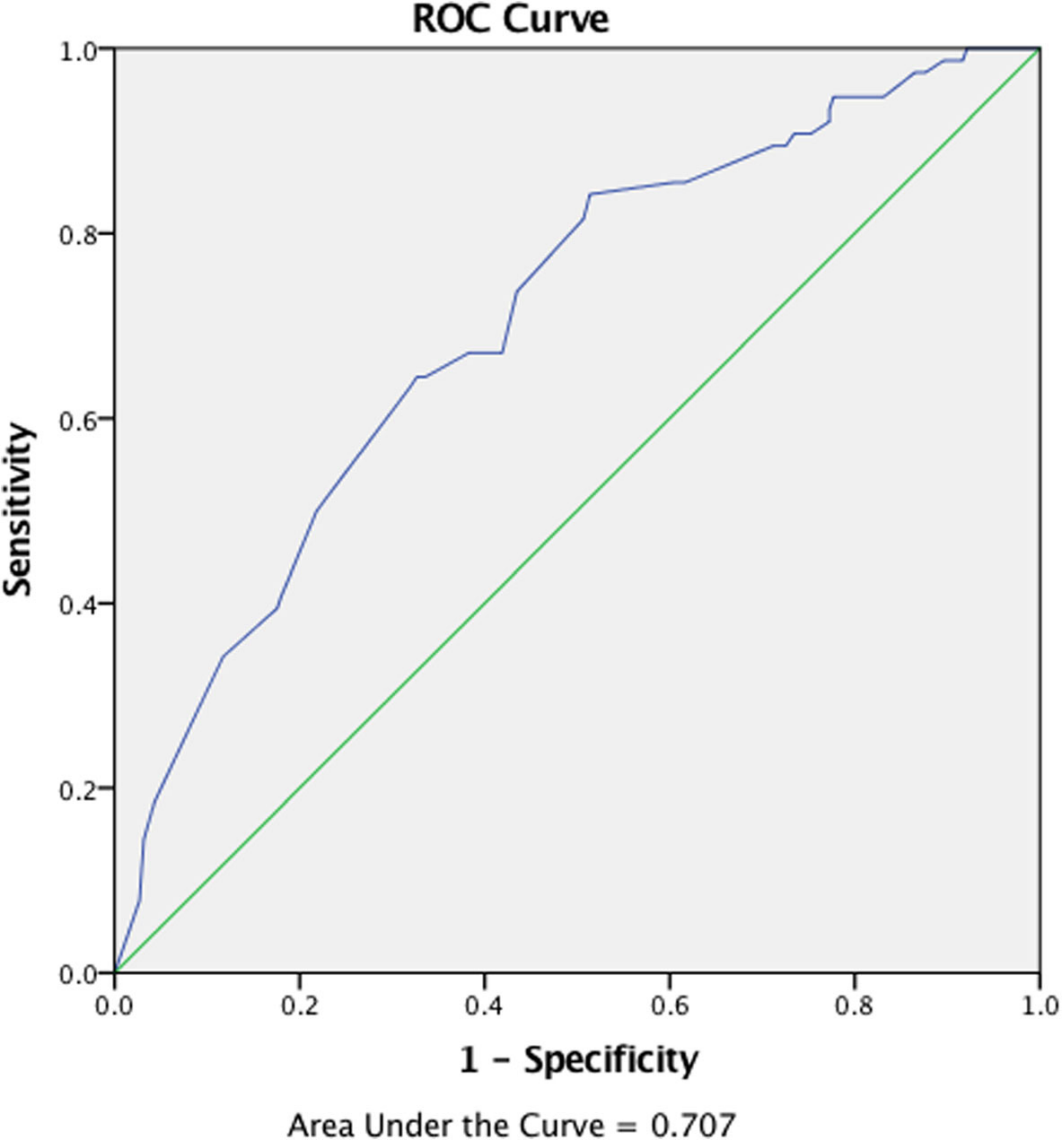
ROC curve of THRI to predict HCC. AUC (area under the curve) = 0.707

A total of 76 patients developed HCC during the follow-up. The BCLC stage, presentation, and following treatment were recorded in Additional file 1 in detail. The overall mean THRI value was 239.2 ± 68.2. The THRI value differed between the HCC group and the non-HCC group (279.5 ± 57.1 vs. 232.3 ± 67.6, *p* < 0.001). The area under the ROC curve of the THRI to predict HCC was 0.707 ([95% CI 0.645-0.769], *p* < 0.001, Fig. 2). Based on the Youden index, the cutoff point was a THRI value of 226, which presented a sensitivity of 0.842, specificity of 0.486, positive predictive value of 0.219, negative predictive value of 0.947, and accuracy of 0.538.

All the patients were stratified into three risk groups based on the THRI score (Table 2). No patients with THRI < 120 (23 patients) progressed to HCC by their last follow-up visit. Of 248 patients in the intermediate-risk group, 20 developed HCC. A total of 249 patients were scored with a THRI value more than 240, and 56 of them developed HCC in the end.

**Table 2:** Risk groups based on THRI score

THRI score	All patients (*n* = 520)	HCC (*n* = 76)	Non-HCC (*n* = 444)
< 120, *n* (%)	23 (4.4)	0 (0.0)	23 (5.2)
120–240, *n* (%)	248 (47.7)	20 (26.3)	228 (51.4)
> 240, *n* (%)	249 (47.9)	56 (73.7)	193 (43.5)

Logistic regression was conducted to analyze the relationship between the THRI and HCC risk (Table 3). THRI was associated with a higher risk of HCC (odds ratio [OR] = 1.015 [95% CI 1.006–1.024]) when comparing the high-risk group with the low-risk group. After the adjustment, the THRI remained associated with HCC risk (*p* = 0.036, OR 1.026 [95% CI 1.002–1.051]).

**Table 3:** Potential factors associated with HCC

High-risk vs. low-risk						
	Univariate			Multivariate		
	*p* value	OR	95% CI	*p* value	OR	95% CI
THRI	*0.001*	*1.015*	*1.006–1.024*	*0.035*	*1.026*	*1.002–1.051*
BMI	0.266	1.063	0.954–1.184	0.778	1.042	0.784–1.383
ALT	0.352	1.001	0.999–1.003	0.714	1.010	0.958–1.065
AST	0.420	1.001	0.998–1.004	0.089	1.045	0.993–1.099
GGT	0.198	0.998	0.994–1.001	0.293	0.994	0.982–1.005
ALB	0.878	1.004	0.956–1.054	0.394	1.097	0.886–1.359
TBil	0.930	1.000	0.991–1.008	0.328	0.960	0.885–1.042
Cr	0.376	0.996	0.987–1.005	0.775	0.990	0.927–1.058
AFP	*0.021*	*1.022*	*1.003–1.041*	0.770	1.004	0.979–1.028
APRI	0.182	1.040	0.982–1.102	0.294	0.540	0.170–1.710
FIB-4	0.099	1.035	0.993–1.079	0.793	1.045	0.753–1.451
MELD	0.794	0.993	0.938–1.050	0.713	0.920	0.589–1.436
Child-Pugh Score	0.714	1.035	0.863–1.241	0.068	4.269	0.896–20.330
Intermediate-risk vs. Low-risk						
	Univariate			Multivariate		
	*p* value	OR	95% CI	*p* value	OR	95% CI
THRI	0.101	1.010	0.998–1.023	0.692	1.012	0.954–1.074
BMI	0.696	1.033	0.877–1.217	0.881	1.041	0.612–1.773
ALT	0.505	0.996	0.986–1.007	0.679	0.972	0.848–1.113
AST	0.458	0.996	0.985–1.007	0.846	1.016	0.869–1.187
GGT	0.549	0.999	0.995–1.002	0.144	1.015	0.995–1.035
ALB	0.992	1.000	0.929–1.077	0.076	1.972	0.931–4.176
TBil	0.520	0.996	0.983–1.009	0.866	0.958	0.580–1.582
Cr	0.588	1.002	0.996–1.007	0.410	0.928	0.777–1.109
AFP	0.922	1.000	0.990–1.009	0.973	1.002	0.907–1.107
APRI	0.390	0.883	0.665–1.173	0.213	0.044	0.000–5.946
FIB-4	0.392	0.958	0.867–1.058	0.105	3.604	0.766–16.965
MELD	0.621	1.020	0.944–1.102	0.814	1.162	0.333–4.051
Child-Pugh Score	0.752	1.048	0.784–1.402	0.158	20.454	0.309–1355.822

*OR* odds ratio, *CI* confidence interval, *THRI* Toronto hepatocellular carcinoma risk index, *BMI* body mass index, *INR* international normalized ratio, *PT* prothrombin time, *ALT* alanine aminotransferase, *AST* aspartate aminotransferase, *GGT* γ-glutamyl transpeptidase, *ALB* albumin, *TBil* total bilirubin, *Cr* creatinine, *AFP* alpha fetoprotein, *APRI* AST to platelet ratio index, *FIB-4* Fibrosis-4 score, *MELD* model for end-stage liver disease

The italicized data were statistically significant

**Fig. 3:**
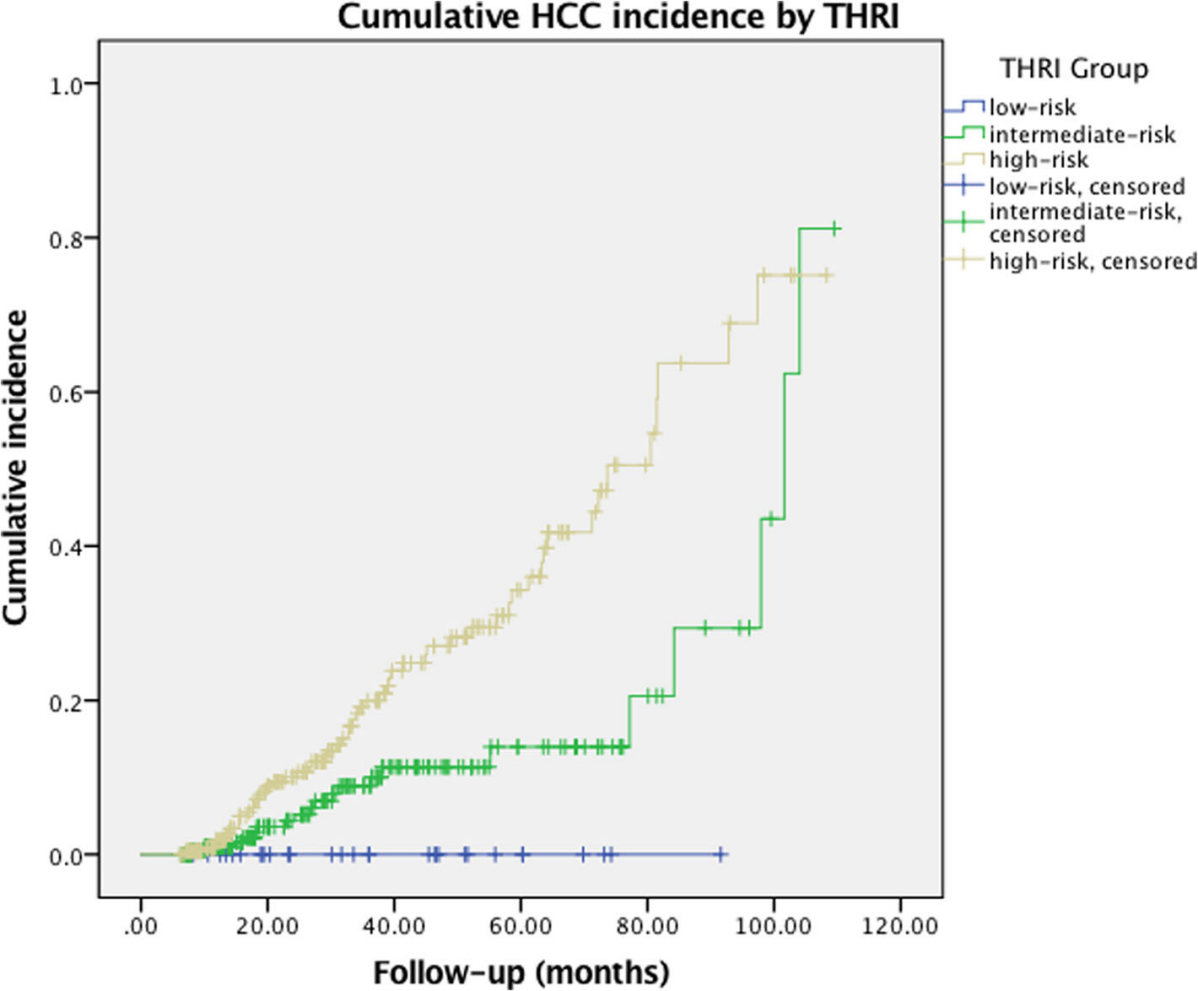
Cumulative incidence of HCC by THRI risk group. The cumulative incidence of HCC is shown for the low-risk group (THRI < 120), intermediate-risk group (THRI 120–240), and high-risk group (THRI > 240). Cumulative incidence was compared between groups using a log-rank test (*p* < 0.001, low-risk vs. intermediate-risk with *p* = 0.191, intermediate-risk vs. high-risk with *p* < 0.001, and low-risk vs. high-risk with *p* = 0.022)

The Kaplan-Meier method was used to calculate the cumulative HCC incidence of each THRI group (Fig. 3). The low-risk group showed no risk of HCC development with both a 5-year and 10-year cumulative HCC incidence of 0%. The high-risk group had a 5-year cumulative incidence of 34%, which was much higher than that of the intermediate-risk group (13%). However, the 10-year cumulative HCC incidence of the intermediate-risk group sharply rose to 81%, exceeding that of the high-risk group (75%). The curves of three risk groups were compared by log-rank test (*p* < 0.001).

## Discussion

In this retrospective cohort study, we first validated the THRI in predicting the development of HCC in Asian patients with cirrhosis. Patients who developed HCC at follow-up presented a higher baseline THRI. The ROC curve supported that the THRI had a good ability to predict HCC with a high sensitivity. Compared with the low-risk group (THRI < 120), the high-risk group (THRI > 240) presented higher odds of developing HCC. Furthermore, the 5-year cumulative HCC incidence of the high-risk group was significantly higher than that of the intermediate-risk group and the low-risk group. This result supported the THRI as a qualified scoring system to predict the development of HCC in patients with cirrhosis.

The 5-year and 10-year cumulative HCC incidence of the intermediate-risk and the high-risk group in this study were much higher than those reported in the study by Sharma et al. [21]. (The 5-year cumulative incidence of the intermediate group was 13% in this study vs. 4% in Sharma’s. The 5-year cumulative incidence of the high-risk group was 34% vs. 15% in Sharma’s. The 10-year cumulative incidence of the intermediate group was 81% in this study vs. 10% in Sharma’s. The 10-year cumulative incidence of the high-risk group was 75% in this study vs. 32% in Sharma’s.) The divergence in the etiology of cirrhosis between two studies might account for that. Patients with CHB were a big component of patients with cirrhosis and HCC in this study. Although the cumulative incidence of HCC for each etiology was not calculated due to the limited sample size, according to the study conducted by Sharma et al., the 10-year cumulative HCC incidence of the patients with CHB was the highest (23.2%). The high proportion of CHB might contribute a lot to the high incidence in this study. The “Other group,” including cirrhosis caused by parasitic infection, was another big component of cirrhosis and HCC, which also potentially influenced the results. The 5-year and 10-year cumulative incidence of low-risk group (both 0%) were lower than those in the study by Sharma et al., (1% and 3%) perhaps due to the small sample size (*n* = 23). Further study with large samples is needed as to that.

The 10-year cumulative HCC incidence of the intermediate-risk group exceeded that of the high-risk group, which led to an intersection of the curves after 100 months in Fig. 3. On one hand, categories of etiology differed obviously between two groups (Additional file 2). In the intermediate-risk group, the “Other group” was the biggest component and was big enough to bring about strong influence. In the high-risk group, HBV infection was the most common cause and most patients with CHB (139/184) in this study were antiviral-treated. According to Abu-Amara M et al., antiviral treatment had an effect on reducing HCC risk [23]. On the other hand, the THRI was more likely to lose its accuracy the longer patients were followed, because other adverse factors such as decompensated cirrhosis, advanced liver diseases, and deterioration of comorbidities, might negatively impact survival and influence the incidence of HCC. Additionally, the longest follow-up was 109.48 months, so it would be appropriate to use the 10-year cumulative incidence as a reference only.

Compared to CHB, CHC has a lower prevalence in China [12]. Before DAAs being listed, PR was the main method for treating HCV-infection. PR can be utilized to all gene-types of HCV, which is much more cost-effective than DAAs. Four of the patients with CHC did not receive antiviral treatment owing to decompensated cirrhosis, which was an absolute contraindication of pegylated interferon-alfa. The others received antiviral treatment discontinuously and failed to achieve SVR. In view of these situations, DAAs are alternative and recommended in WHO guideline for people with hepatitis C infection [24] to shorten the course of treatment and improve tolerance and SVR rate. We hope that after clinical trials, more kinds of DAAs can be listed on the Chinese market.

When comparing the high-risk group with the low-risk group in the logistic regression, the value of AFP was not promising for predicting HCC. AFP at high levels (> 500 ng/mL) was diagnostic, but AFP alone was not recommended for routine screening of HCC because of its low specificity [10]. This result reminded us to be skeptical of using this value in diagnosing HCC because increasing levels of AFP can be triggered by active hepatitis and cirrhosis.

No significant differences were found in some clinical parameters between patients who developed HCC and patients who did not. The mean age of patients was 61 ± 13 years, but comorbidities and complications were too common to influence physical and clinical parameters. Additionally, other confounders need to be considered. Obesity was defined as BMI > 30 and HCC risk increased in obese patients [25, 26]. In this study, it is difficult to distinguish “obesity” caused by massive ascites from obesity caused by BMI, so the comparison might be somewhat influenced.

In addition to the four components of the THRI, previous studies revealed that multiple factors, including obesity, diabetes/insulin resistance, high alcohol intake, smoking history, ethnicity (African and Asian family origin), duration of infection, patients with genotype C and core promoter mutants, HBV-DNA level, and elevated ALT, increased the risk of developing HCC [6, 17, 27–32]. Multiple scoring systems and models comprised of these factors have been used to predict the risk of HCC; however, these findings were mainly summarized from studies in high prevalence areas of hepatitis virus infection with limitations mentioned before.

Survival for patients with HCC was very low, with a 5-year relative survival rate of only 18% from 2005 to 2011 in the USA [33]. Due to economics, imperfect surveillance programs and other reasons, patients’ adherence to screening for HCC is poor [28, 34–37]. THRI uses commonly available variables and can be easily calculated, which is useful in the risk stratification in cirrhotic populations and in the surveillance of HCC. By identifying high-risk populations, potentially curative treatments can be applied to them. The 5-year and 10-year cumulative HCC incidence of the low-risk group were very low (< 5%) in this study and the study conducted by Sharma et al. Thus, it may not be necessary to conduct biannual surveillance as recommended for low-risk populations, considering THRI is cost-effective and totally enough for screening. We recommend that THRI be used to patients when cirrhosis is diagnosed. It would be reasonable to instruct surveillance programs with THRI in the future. Of course, before THRI is widely utilized to geographically diverse patients, it requires more validation.

There were some limitations in this study. This validation was conducted using a single-center design, which might cause selection bias. In this retrospective study, missing data were inevitable and led to limited sample size during the selection of participants. The limited sample size influenced the statistical results to some extent and restricted the analysis of HCC incidence in different etiologies. Larger-sampling sizes and multicenter prospective studies are needed in the future.

## Conclusions

The THRI was first validated in Rotterdam, Netherlands, when being developed and showed good predictive ability [21]. By conducting this study, we validated the potential of the THRI as a qualified scoring system to identify high-risk patients with cirrhosis developing HCC in Asia. Therefore, the THRI may play an important role in secondary prevention for HCC.

## Additional files

Additional file 1: Characteristics of 76 patients with HCC. (XLSX 11 kb)

Additional file 2: Categories of etiology in three risk groups. (XLSX 9 kb)

## Abbreviations

AASLD: American Association for the Study of Liver Diseases
ADRESS: Age, diabetes, race, etiology of cirrhosis, sex and severity of liver dysfunction
AFP: Alpha-fetoprotein
AIH: Autoimmune hepatitis
ALD: Alcoholic liver disease
ALT: Alanine transaminase
APASL: Asian Pacific Association for the Study of the Liver
APRI: AST to platelet ratio index
AST: Aspartate aminotransferase
BMI: Body mass index
CHB: Chronic hepatitis B
CHC: Chronic hepatitis C
CI: Confidence interval
CUHK: Chinese University of Hong Kong
DAAs: Direct-acting antiviral agents
EOB-MRI: Gd-EOB-DTPA-enhanced MRI
FIB-4: Fibrosis-4
GAG-HCC: Guide with Age, Gender, HBV-DNA, Core Promoter Mutations and Cirrhosis
Gd-EOB-DTPA: Gadolinium ethoxybenzyl diethylenetriamine pentaacetic acid
HALT-C: Hepatitis C Antiviral Long-Term treatment against Cirrhosis
HBV: Hepatitis B Virus
HCC: Hepatocellular carcinoma
HCV: Hepatitis C Virus
INR: International normalized ratio
IQR: Interquartile range
MELD: Model for end-stage liver disease
NAFLD: Non-alcoholic fatty liver disease
OR: Odds ratio
PBC: Primary biliary cirrhosis
PR: Pegylated interferon-alfa plus Ribavirin
PSC: Primary sclerosing cholangitis
REACH-B: Risk Estimation for Hepatocellular Carcinoma in Chronic Hepatitis B
REVEAL-HBV: Risk Evaluation of Viral Load Elevation and Associated Liver Disease/Cancer-Hepatitis B Virus
ROC: Receiver operator characteristic
SD: Standard deviation
THRI: Toronto hepatocellular carcinoma risk index
